# Gd(III)-induced Supramolecular Hydrogelation with Enhanced Magnetic Resonance Performance for Enzyme Detection

**DOI:** 10.1038/srep40172

**Published:** 2017-01-11

**Authors:** Yongquan Hua, Guojuan Pu, Caiwen Ou, Xiaoli Zhang, Ling Wang, Jiangtao Sun, Zhimou Yang, Minsheng Chen

**Affiliations:** 1 Department of Cardiology, Zhujiang Hospital of Southern Medical University, Guangzhou 510280, P. R. China; 2 School of Pharmaceutical Engineering & Life Science, Changzhou University, Changzhou 213164, P. R. China; 3 State Key Laboratory of Medicinal Chemical Biology, Key Laboratory of Bioactive Materials, Ministry of Education, College of Life Sciences, Nankai University, and Collaborative Innovation Center of Chemical Science and Engineering, Tianjin 300071, P. R. China

## Abstract

Here we report a supramolecular hydrogel based on Gd(III)-peptide complexes with dramatically enhanced magnetic resonance (MR) performance. The hydrogelations were formed by adding Gd(III) ion to the nanofiber dispersion of self-assembling peptides naphthalene-Gly-Phe-Phe-Tyr-Gly-Arg-Gly-Asp (Nap-GFFYGRGD) or naphthalene-Gly-Phe-Phe-Tyr-Gly-Arg-Gly-Glu (Nap-GFFYGRGE). We further showed that, by adjusting the molar ratio between Gd(III) and the corresponding peptide, the mechanical property of resulting gels could be fine-tuned. The longitudinal relaxivity (r_1_) of the Nap-GFFYGRGE-Gd(III) was 58.9 mM^−1^ S^−1^, which to our knowledge is the highest value for such peptide-Gd(III) complexes so far. Such an enhancement of r_1_ value could be applied for enzyme detection in aqueous solutions and cell lysates.

The interaction of metal ions with amino acid side chains in proteins prevails in nature, and such interaction is crucial to the biological functions of proteins^1^,^2^. Inspired by nature, researchers have developed many biofunctional nanomaterials formed by the specific interactions between metal ions and peptides^3^,^4^,^5^,^6^,^7^. Among them, supramolecular hydrogels^8^,^9^,^10^,^11^,^12^, especially those of peptides^13^,^14^,^15^,^16^,^17^,^18^,^19^,^20^,^21^,^22^,^23^,^24^,^25^, have attracted research interests due to their inherent biocompatibility, degradability, and fast responsiveness to external stimuli^26^,^27^,^28^,^29^,^30^,^31^,^32^. Successful examples of metal ion-induced supramolecular hydrogelations have been reported. For example, Scheneider and co-workers reported on the zinc-induced formation of a hydrogel that was promising for wound healing^33^. Xu and Stupp had also used potassium and calcium ions, respectively, to induce the formation of supramolecular hydrogels with adjustable mechanical properties^34^,^35^. We recently reported copper ion-induced formation of nanofibers and hydrogels with quenched fluorescence property^36^. These works highlight the potential of metal ion-triggered supramolecular hydrogels in cell culture, tissue engineering, sensing, and drug delivery.

Magnetic resonance imaging (MRI) is a powerful, non-invasive diagnostic technology with high spatial resolution. MRI contrast agents, such as Gd(III) complexes, are often used to enhance the contrast^37^ between pathological and normal tissues by altering the longitudinal and transverse (T_1_ and T_2_) relaxation times. The relaxivity of a contrast agent is dependent on several factors, including the number of water molecules in the coordination shell, the exchange rate of the coordinated water with the bulk water, and the tumbling rate of the complex in solution^38^. However, the majority of Gd(III) complexes used as MRI contrast agents in clinical practice, such as Magnevist (DOTA(Gd)), do not display optimal relaxivity due to small sizes and rapid tumbling in solution. To improve relaxivity, the small molecular contrast agents were covalently or noncovalently bound to macromolecules such as polymers^39^, dendrimers^40^, carbohydrates^41^, liposomes^42^, and proteins^43^. In this respect, our goal is to develop a supramolecular hydrogel based on Gd(III)-peptide complexes with optimized relaxivity.

## Results and Discussion

**Figure 1:**
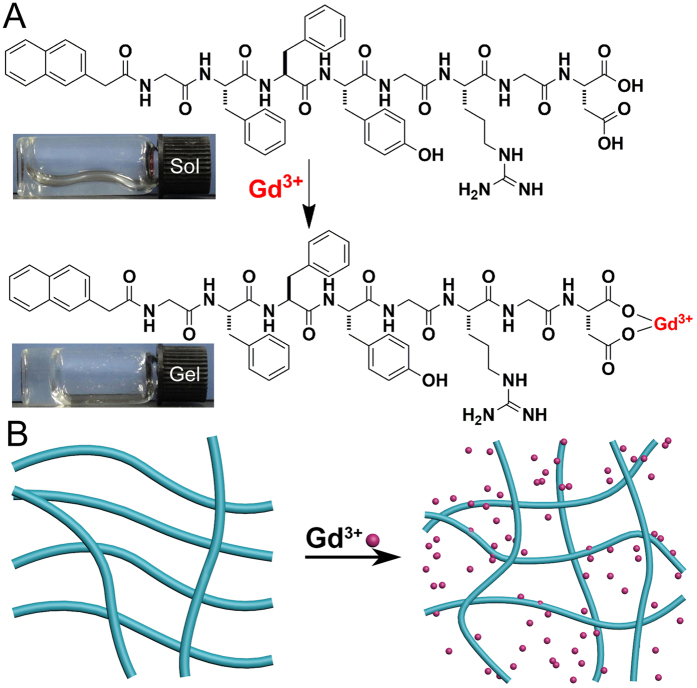
(**A**) The chemical structures of Nap-GFFYGRGD and possible Gd^3+^-peptide complex (Insert: optical images of a HEPES solution of Nap-GFFYGRGD (0.25 wt%, pH = 7.4) with or without 0.33 equiv. of Gd(III)) and (**B**) The schematic illustration for hydrogel formation.

We opted to use Gd(III) to trigger the formation of supramolecular nanofibers and hydrogels, because we believed that the formation of supramolecular nanostructures of Gd(III) complex might improve the magnetic resonance (MR) performance of the MR contrast agent of Gd(III). We first chose one of our recently developed self-assembling peptides, Nap-GFFYGRGD for the test (Fig. 1A). Similar to many self-assembling peptide molecules that can form supramolecular nanostructures, the driving force of the self-assembly of the Nap-GFFYGRGD is hydrogen bond between peptide chain and π -π interaction between aromatic groups on Nap and amino acid of F. The peptide Nap-GFFYGRGD could self-assemble into supramolecular nanofibers but not hydrogels at the concentrations lower than 0.7 wt% in 4-(2-Hydroxyethyl)-1-piperazineethanesulfonic acid (HEPES, pH = 7.4) solution^44^. The reason for the peptide incapable of forming hydrogels at these concentrations was the weak inter-fiber interaction and repulsion between negatively charged fibers. We hypothesized that, if we could enhance the inter-fiber interaction or decrease the repulsion force between fibers, we might obtain stable three dimensional (3D) network of nanofibers and supramolecular hydrogels (Fig. 1B). There were two carboxylic acid residues on the terminal amino acid (aspartic acid (D)) of the peptide that might form complex with metal ions such as Gd(III) (Fig. 1A). We therefore planned to test whether the addition of Gd(III) would cross-link the nanofibers and trigger the formation of supramolecular hydrogels of the peptide.

**Figure 2:**
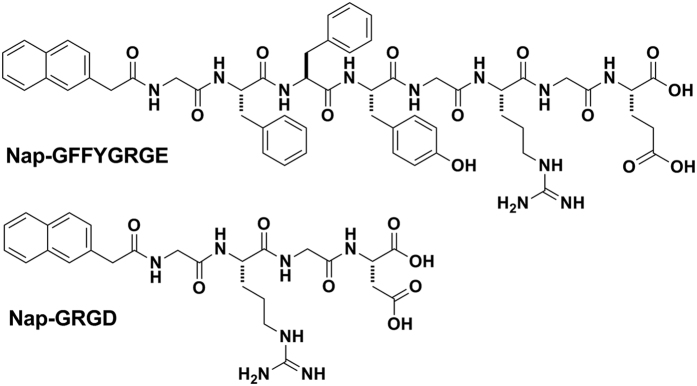
The chemical structures of Nap-GFFYGRGE and Nap-GRGD.

We fixed the peptide concentration to be 0.25 wt% (2.5 mg/mL) in the HEPES solution for the test. The peptide formed a solution at this concentration, and the addition of 0.33 mole equivalent of Gd(III) to the peptide into the solution could indeed trigger the supramolecular hydrogel formation (Fig. 1A) within 10 seconds. The minimum equiv. of Gd(III) needed for gelation was 0.05 (Supplementary Fig. S7). However, it took a longer time (about 16 h) for the formation of a hydrogel. In addition, it took about 10 h and 6 h to form the hydrogels with the addition of 0.1 and 0.2 equiv. of Gd(III) into the peptide (0.25 wt%) solution, respectively. It suggested that the gelation time decreased with increasing the Gd(III) concentration. The diameter of nanofibers in the gel would become smaller with the increasing Gd(III) concentration as well. The short-peptide hydrogels are different with polymeric hydrogels. They are soft enough to be filtered. Therefore we used the filtration to remove the nanofibers. If passing the resulting gel through a filter (0.45 μm), it was found that less than 1.0% of Gd(III) remained in the filtrate (determined by Inductively Coupled Plasma (ICP)). In the meantime, we also found that 90.2% of the Nap-GFFYGRGD converted to the hydrogel by calculating the residual amount of the peptide in the filtrate. This observation indicated that the Gd(III) could form a tight complex with the peptide. Several pioneering results had demonstrated that one Gd(III) could form a complex with three carboxylic acids^25^. We therefore hypothesized that Gd(III) served as a cross-linker to enhance the inter-fiber interaction of nanofibers of Nap-GFFYGRGD, resulting in supramolecular hydrogelations (Fig. 1B). The result was similar to our previous report that a recombinant protein with four binding sites could serve as a cross-linker to trigger hydrogelations of peptide nanofiber solutions^45^. We then introduced the peptide naphthalene-Gly-Phe-Phe-Tyr-Gly-Arg-Gly-Glu (Nap-GFFYGRGE) and naphthalene-Gly-Arg-Gly-Asp (Nap-GRGD) (Fig. 2) as the control to justify that the Gd(III) could serve as a cross-linker to enhance the inter-fiber interactions in the peptide solutions and lead to the hydrogelation. The peptide Nap-GFFYGRGE has the same self-assembly motif, Nap-GFFY, as the Nap-GFFYGRGD, while the Nap-GRGD doesn’t have. The critical difference between Nap-GFFGRGD and Nap-GFFYGRGE is the last amino acid residue. The former is aspartic acid (D), the latter is glutamic acid (E). The addition of 1 equiv. of Gd(III) to the HEPES solution of the peptide Nap-GRGD (1.0 wt%) did not lead to hydrogelations, because it could not firstly self-assemble into nanofibers. Hydrogelations could also be observed by adding 0.33 equiv. of Gd(III) to the HEPES solution of Nap-GFFYGRGE (0.25 wt%, Supplementary Fig. S8) because the peptide Nap-GFFYGRGE could also form a nanofiber dispersion at this concentration (Supplementary Fig. S11A). These observations further suggested that the hydrogelations were due to the enhancement of inter-fiber interactions. Another possible reason for hydrogelation was the charge screening of fibers because the Gd(III) was positively charged and the fibers were negatively charged.

**Figure 3:**
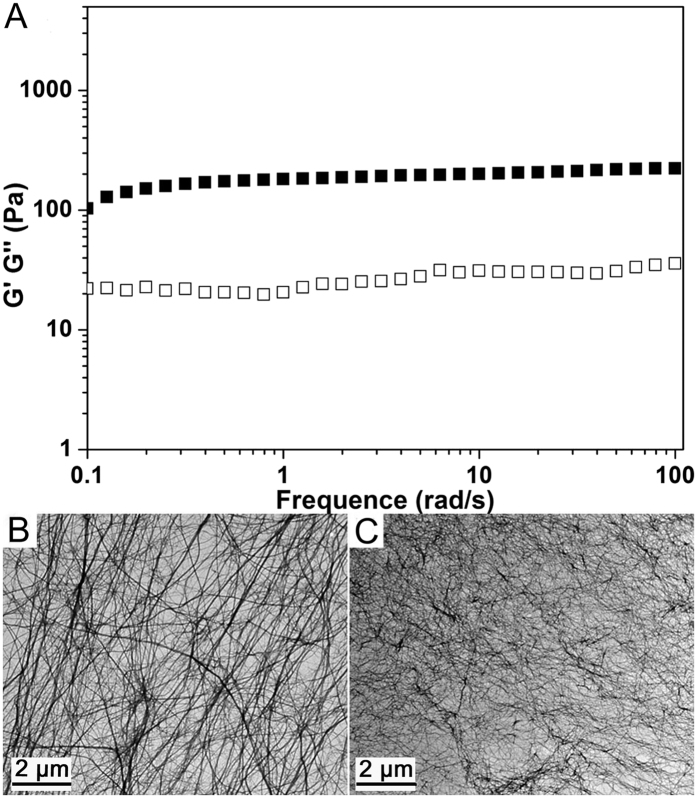
(**A**) Dynamic frequency sweep at the strain of 1% of the gel in Fig. 1 and TEM images of (**B**) a HEPES solution of Nap-GFFYGRGD (0.25 wt%) and (**C**) the resulting gel in the presence of 0.33 equiv. of Gd(III).

Afterward we performed rheological measurements to study the mechanical properties of the hydrogel containing 0.25 wt% of Nap-GFFYGRGD and 0.33 equiv. of Gd(III). The result in Fig. 3A showed that both the storage moduli (G’) and the loss storage (G”) values exhibited weak frequency dependences at the range of 0.1 to 100 rad/s. The G’ value of the gel was about 200 Pa and it was about an order of magnitude bigger than its G” value. These observations suggested the formation of a true hydrogel^46^. The gels formed with higher amounts of Gd(III) exhibited slightly bigger G’ values (Supplementary Figs S9 and S10), which was consistent with results from Xu and Stupp groups that increasing the concentration of metal ions led to better mechanical property of the gels^33^. The transmission electron microscopy (TEM) was also used to characterize the nanostructures in the solution and the gel. As shown in Fig. 3B, uniform nanofibers with the diameter of 30–50 nm was observed for the HEPES solution of the peptide, which was consistent with our previous observations^36^. Upon the addition of 0.33 equiv. of Gd(III) into the peptide solution, the diameter of the nanofibers became smaller (20–35 nm, Fig. 3C). The density of cross-linking points of the nanofibers in the gel was much higher than that in the peptide nanofiber solution, which was consistent with our recent observations in hydrogels formed by adding cis-dichlorodiamineplatinum(II) (DDP) to a taxol-peptide amphiphile solution^47^. Similar to our previous report^36^, the metal ions chelated with the peptide molecules by forming complex with two intra-molecular carboxylic acids on the same peptide molecules. With increasing amount of metal ions, more peptide molecules dissociated from the nanofibers to form complex with the metal ions and the complex re-assembled into nanofibers with smaller diameters. The formation of Gd(III)-peptide complex decreased the inter-fiber repulsion, leading to the formation of stable 3D fiber networks and hydrogels.

**Figure 4:**
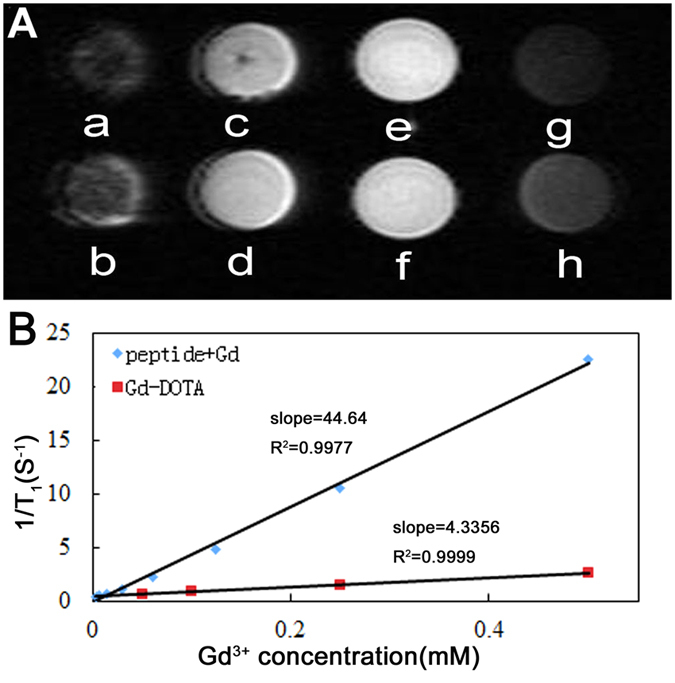
(**A**) T_1_ map of samples of HEPES solutions containing 125 μM of Gd(III) and (a) 1 equiv., (b) 2 equiv., (c) 3 equiv., (d) 4 equiv., (e) 5 equiv., (f) 6 equiv. of the peptide, (g) without peptide, and (h) the HEPES solution containing 125 μM of DOTA(Gd) (Magnevist), (**B**) Plot of relaxation rate r_1_ versus Gd^3+^ concentration for peptide-Gd(III) complex (Nap-GFFYGRGD: Gd(III) = 6:1, HEPES solution, pH = 7.4) and the Magnevist. The relaxivity value r_1_ was obtained from the slope of linear fitting of the experimental data.

We further investigated whether the hydrogelation could improve the MR performance of Gd(III) with a 1.2 T MRI scanner. Figure 4A showed the T_1_-weighted MR images of HEPES solutions of Gd(III) with different equiv. of the peptide and the HEPES solution of clinically used Magnevist (DOTA(Gd)). The results clearly indicated that, for the Gd(III) solutions with different amounts of the peptide, their MR signal enhanced with the increase of the peptide concentrations. Such enhancement reached the equilibrium when the equiv. of peptide was more than 4. When the equiv. of the peptide was bigger than 3, the peptide-Gd(III) complexes exhibited big contrast enhancements and therefore we observed much brighter images from these samples (Fig. 4A-c,d,e, and f) than the sample of Magnevist (Fig. 4A-h) and the sample of Gd(III) without the peptide (Fig. 4A-g). We then quantified the MR signal enhancement by calculating the r_1_ value. As shown in Fig. 4B, the r_1_ value of Magnevist was about 4.3 mM^−1^ S^−1^, while this value for our peptide-Gd(III) complex (peptide: Gd(III) = 6: 1) was 44.6 mM^−1^ S^−1^. Similar observations were obtained for Nap-GFFYGRGE-Gd(III) complex, and its r_1_ value was 58.9 mM^−1^ S^−1^ (Supplementary Fig. S14). The r_1_ value of our peptide-Gd(III) complexes (Nap-GFFYGRGD-Gd(III) and Nap-GFFYGRGE-Gd(III) complexes) was about 10.4 and 13.7 times that of Magnevist. To the best of our knowledge, these values were the biggest ones for peptide-Gd(III) complexes^3^,^42^,^43^,^48^,^49^,^50^. Such a huge enhancement was due to the binding of Gd(III) to the nanofibers and the formation of complexes between Gd(III) and the peptides. The formation of complexes dramatically slowed the molecular tumbling of Gd(III) and reduced the relaxation time of water molecules binding to the Gd(III), thus leading to the huge enhancement of the r_1_ value. We thought that amino acid E possessed one more CH_2_ between peptide backbone and the side chained carboxylic acid. Therefore E is more flexible and it might be more easily to form a complex with Gd(III), which explained why the r_1_ value of Nap-GFFYGRGE was higher than that of Nap-GFFYGRGD.

**Figure 5:**
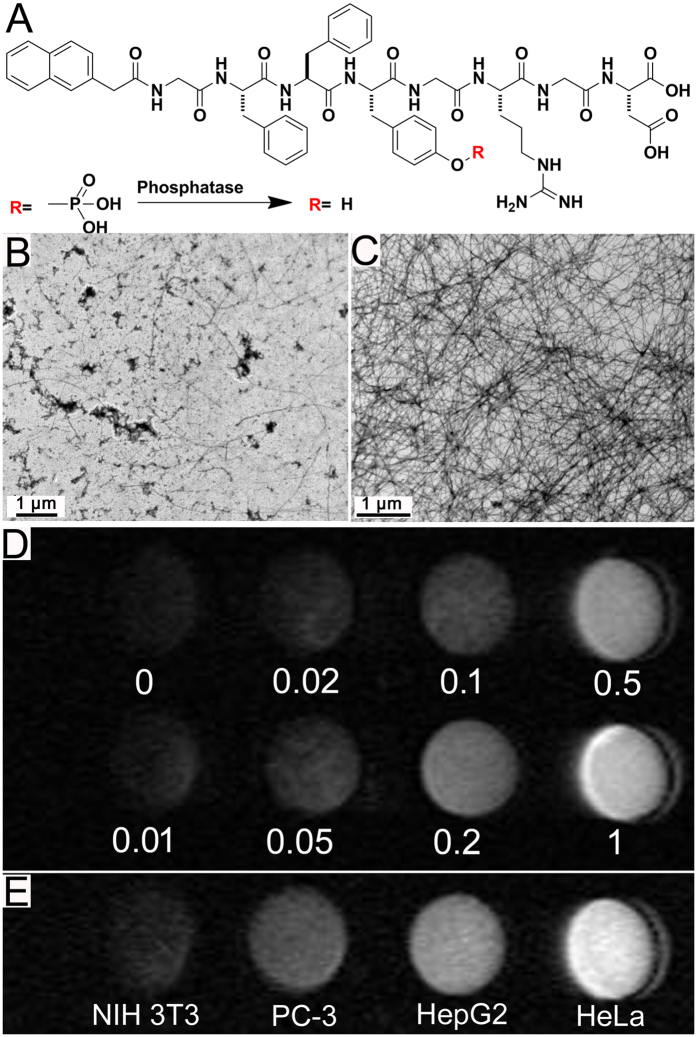
(**A**) The chemical structure of Nap-GFFpYGRGD and its enzymatic transformation by phosphatase, TEM images of a HEPES solution containing 125 μM of Gd(III) and 6 equiv. of Nap-GFFpYGRGD (750 μM) (**B**) without or (**C**) with phosphatase, T_1_ map of HEPES solutions containing 125 μM of Gd(III) and 6 equiv. of Nap-GFFpYGRGD incubated with (**D**) different concentrations of phosphatase (U/mL) or (**E**) different cell lines (lysates from 2 * 10^4^ cells) for 1 h at room temperature.

Supramolecular hydrogelations have been extensively applied for the detection of important analytes by naked eyes in complex fluids^51^,^52^,^53^,^54^,^55^. We envisioned that the formation of Gd(III)-peptide complex with dramatically enhanced MR performance could also be used to detect enzyme activity in complex samples. We therefore designed and synthesized a peptide naphthalene-Gly-Phe -Phe-Tyr(phosphate)-Gly-Arg-Gly-Asp (Nap-GFFpYGRGD) (Fig. 5A). We assumed that the peptide Nap-GFFpYGRGD could not self-assemble into nanofibers due to the presence of highly hydrophilic phosphoric acid residue on pY. However, the peptide could be converted to Nap-GFFYGRGD by the enzyme of alkali phosphatase (ALP), which could self-assemble into nanofibers and form complexes with Gd(III). We used TEM to characterize the nanostructures of peptide solution before and after the addition of ALP in the presence of Gd(III). As shown in Fig. 5B, we observed few nanofibers in the HEPES buffer solution containing 125 μM of Gd(III) and 750 μM of Nap-GFFpYGRGD (6 equiv. to Gd(III)). Upon the addition of ALP for 1 h, more than 95% of Nap-GFFpYGRGD was converted to Nap-GFFYGRGD (Supplementary Fig. S16), and we observed high density of nanofibers in the solution (Fig. 5C).

We then tested whether the Nap-GFFpYGRGD-Gd(III) system could be applied to detect ALP activity. Different concentrations of ALP (0–1 U/mL) were added to the HEPES solutions containing 125 μM of Gd(III) and 6 equiv. of Nap-GFFpYGRGD (750 μM). The solutions were then incubated for 1 h at room temperature (20–25 °C) before scanning by a 1.2 T MRI scanner. As shown in Fig. 5D, we obtained a dark image from the sample without ALP and observed more and more bright images from samples with more and more amounts of enzyme. These observations clearly suggested that our system could be used to detect enzyme concentration. It is well known that different cell lines express different levels of phosphatase and cancer cells have much higher expression level of phosphatase than normal cells. It is of great importance to detect the expression level of phosphatase in different cell lines. We therefore tested whether our strategy could be applied for the detection of enzyme activity in the complex sample of cell lysates. Different kinds of cell lysates (HeLa, HepG2, PC-3, and NIH 3T3) were prepared and added into the HEPES solution containing Gd(III) and Nap-GFFpYGRGD. After incubation for 1 h at room temperature, we captured the T_1_-weighted MR images by a 1.2 T MRI scanner. As shown in Fig. 5E, compared to the image from normal cell lysate (3T3), those from cancer cell lysates were much brighter, suggesting the higher expression levels of phosphatase in cancer cells. Among samples of cancer cells, the one containing HeLa cell lysate showed the brightest image, indicating the highest expression level of phosphatase in HeLa cells. These observations suggested that our method could be applied for direct detection of enzyme activity in complex fluids such as cell lysates.

## Conclusion

In summary, we have successfully used the MR contrast agent Gd(III) to trigger the formation of supramolecular hydrogels of self-assembling peptides. The Gd(III) could form tight complexes with the peptide, resulting in reducing the relaxation times of water molecules and a huge enhancement of r_1_ value of Gd(III). The hydrogelations and the enhancement of MR signal were instant after simply mixing the peptide and Gd(III) in aqueous solutions. The huge enhancement of MR performance of Gd(III) after chelating with the peptide could be applied for the detection of enzyme activity in both buffer solutions and cell lysates. One of shortcomings of our system is probably the leakage of Gd(III) after the peptide digestion *in vivo*, which may cause toxicity and hinder the *in vivo* application of our system at high concentrations. This shortcoming might be overcome by attaching DOTA(Gd) to the self-assembling peptide nanofibers.

## Methods Summary

### Chemicals

Fmoc-amino acids were obtained from GL Biochem (Shanghai). Naphthalene acetic acid and Gadolinium (III) chloride hexahydrate were purchased from Aladdin Chemistry CO. Ltd (Shanghai). 2-Cl-trityl chloride resin was obtained from Nankai University Resin Co. Ltd. Commercially available reagents were used without further purification, unless noted otherwise. Ultrapure water (produced by a Mili-Q-A10 Advantage Ultrapure Water Purification System, America) was used for all experiments. All other chemicals were reagent grade or better.

### General methods

The synthesized compounds were characterized using ^1^H NMR (Bruker ARX 400, Germany). The LC-MS spectrometric analyses were performed at the Thermo Finnigan LCQ AD System (America). HPLC was conducted at LUMTECH HPLC (Germany) system using a C18 RP column with MeOH (0.1% of Trifluoroacetic acid (TFA), 1 μl/L) and water (0.1% of TFA, 1 μl/L) as the eluents. Rheology was performed on an AR 2000ex (TA instrument, America) system using a parallel plates (40 mm) at the gap of 500 μm. TEM was done on a Tecnai G2 F20 system, operating at 200 kV. The MR and relaxation time was measured by 1.2 T magnetic field (NightOWLIILB985, Germany).

### Peptide synthesis

The peptide was prepared by solid-phase peptide synthesis (SPPS) using 2-chlorotrityl chloride resin and the corresponding N-Fmoc protected amino acids with side chains properly protected by a tert-butyl group or Pbf group or Boc group. After the first amino acid was loaded on the resin by its C-terminal, 20% piperidine in anhydrous N, N’-dimethylformamide (DMF) was used to de-protect Fmoc group. Then the next Fmoc protected amino acid was coupled to the free amino group using O-(benzotriazol-1-yl)-N,N,N’,N’-tetramethyluranium- hexafluorophosphate (HBTU) as the coupling reagent and diisopropylethylamine (DIEA) as catalytic reagent. The growth of the peptide chain was according to the established Fmoc SPPS protocol. After the last amino acid was coupled, excessive reagents were removed by a single DMF wash for 5 min (5 mL per gram of resin), followed by 5 times dichloromethane (DCM) wash for 2 min (5 mL per gram of resin). The peptide was cleaved using 95% of TFA with 2.5% of trimethylsilane (TMS) and 2.5% of H_2_O for 30 min. TFA was removed by rotary evaporator, then 20 mL of ice-cold diethylether was added. The resulting precipitate was filtrated and washed by ice-cold diethylether. The resulting solid was further purified by HPLC and dried by a lyophilizer.

### Nap-GFFYGRGD

^1^H NMR (400 MHz, DMSO-d6) δ 9.19 (s, 1 H), 8.29–8.17 (m, 4 H), 8.13 (d, J = 6.8 Hz, 2 H), 8.09–8.02 (m, 2 H), 7.89–7.79 (m, 3 H), 7.75 (s, 1 H), 7.69 (s, 1 H), 7.48 (dd, J = 8.9, 5.8 Hz, 2 H), 7.42 (d, J = 8.6 Hz, 2 H), 7.25–7.10 (m, 12 H), 7.06 (d, J = 8.3 Hz, 2 H), 6.66 (d, J = 8.3 Hz, 2 H), 4.57–4.41 (m, 4 H), 4.31 (d, J = 5.8 Hz, 1 H), 3.74 (dd, J = 16.3, 10.9 Hz, 5 H), 3.65–3.54 (m, 3 H), 3.10 (d, J = 5.7 Hz, 2 H), 2.94 (ddd, J = 17.6, 16.4, 6.3 Hz, 3 H), 2.83–2.73 (m, 2 H), 2.72–2.64 (m, 2 H), 2.60 (dd, J = 16.7, 6.6 Hz, 1 H), 1.80–1.67 (m, 1 H), 1.52 (dd, J = 13.7, 8.5 Hz, 3 H). MS: calc. M^+^ = 1086.15, obsd. M^+^ = 1086.70.

### Nap-GRGD

^1^H NMR (400 MHz, DMSO-d6) δ 8.33 (t, J = 5.6 Hz, 1 H), 8.23 (dd, J = 15.3, 6.9 Hz, 2 H), 8.11 (d, J = 7.8 Hz, 1 H), 7.97–7.81 (m, 3 H), 7.78 (s, 1 H), 7.48 (td, J = 12.5, 7.2 Hz, 4 H), 7.25 (s, 1 H), 7.12 (s, 1 H), 7.00 (s, 1 H), 4.55 (dd, J = 13.8, 6.5 Hz, 1 H), 4.29 (t, J = 7.3 Hz, 1 H), 3.78 (ddd, J = 17.1, 14.1, 5.7 Hz, 4 H), 3.67 (s, 2 H), 3.07 (d, J = 5.9 Hz, 2 H), 2.89 (s, 1 H), 2.75–2.53 (m, 3 H), 1.76–1.61 (m, 1 H), 1.59–1.35 (m, 3 H). MS: calc. M^+^ = 571.23, obsd.[M + H]^+^ = 572.45.

### Nap-GFFpYGRGD

^1^H NMR (400 MHz, DMSO) δ 8.52 (d, J = 9.1 Hz, 1 H), 8.33 (d, J = 7.6 Hz, 1 H), 8.18 (d, J = 8.2 Hz, 2 H), 8.06 (d, J = 8.5 Hz, 1 H), 7.21 (dt, J = 37.8, 15.0 Hz, 10 H), 7.09 (t, J = 7.5 Hz, 2 H), 6.42 (d, J = 9.2 Hz, 1 H), 4.57 (s, 1 H), 4.52–4.38 (m, 2 H), 3.63 (ddd, J = 22.6, 16.7, 8.4 Hz, 5 H), 3.04 (d, J = 13.7 Hz, 2 H), 2.99–2.90 (m, 2 H), 2.83–2.57 (m, 4 H), 2.55 (s, 2 H). MS: calc. M^+^ = 1166.13, obsd. [M + H]^+^ = 1167.55.

### Nap-GFFYGRGE

^1^H NMR (400 MHz, DMSO-d6) δ 9.22 (s, 2 H), 8.32 (s, 1 H), 8.30 (d, J = 5.3 Hz, 1 H), 8.21 (s, 1 H), 8.16 (s, 2 H), 8.13 (s, 2 H), 8.12–8.08 (m, 4 H), 7.88 (s, 1 H), 7.86 (s, 1 H), 7.83 (s, 3 H), 7.81 (s, 1 H), 7.75 (s, 2 H), 7.56 (s, 1 H), 7.48 (d, J = 3.2 Hz, 1 H), 7.47 (s, 1 H), 7.42 (d, J = 8.4 Hz, 2 H), 7.21 (s, 1 H), 7.20 (s, 3 H), 7.18 (s, 1 H), 7.16 (s, 4 H), 7.07 (s, 1 H), 7.05 (s, 1 H), 6.66 (s, 1 H), 6.64 (s, 1 H), 4.49–4.45 (m, 3 H), 4.29 (d, J = 6.6 Hz, 1 H), 4.22 (d, J = 5.0 Hz, 1 H), 3.75 (s, 6 H), 3.09 (d, J = 5.9 Hz, 2 H), 2.92 (d, J = 13.7 Hz, 4 H), 2.75 (s, 2 H), 2.69–2.63 (m, 2 H), 2.25 (s, 1 H), 1.98–1.94 (m, 1 H), 1.79–1.72 (m, 2 H), 1.54–1.49 (m, 3 H). MS: calc. M^+^ = 1099.47, obsd.[M + 2 H]^+^ = 1101.40.

### Formation of the hydrogels

In order to investigate the hydrogel formation, the Nap-GFFYGRGD was first dissolved in a small amount of HEPES in a little glass bottle (as shown in Supplementary Fig. S7), then about 2 equiv. of sodium hydroxide solution (1 M) were added to adjust the pH to about 7.4. Afterward, a certain amount of HEPES was supplemented to obtain Nap-GFFYGRGD solution with desired concentration. Then HEPES solution (pH = 7.4) containing different equivalents (0.02, 0.05, 0.1, 0.2, 0.33) of Gd(III) was added into the above solution to initial the hydrogelation. The similar procedure were conducted when the hydrogels of Nap-GFFYGRGE were prepared.

### Rheology

Rheology test was carried out on an AR 2000ex (TA instrument) system, 40 mm parallel plates was used during the experiment at the gap of 500 μm. For the dynamic time sweep, the solution of compounds were directly transferred to the rheometer and it was conducted at the frequency of 1 rad/s and the strain of 1% immediately. The gel was also characterized by the mode of dynamic frequency sweep in the region of 0.1–100 rad/s at the strain of 1%. A dynamic strain sweep at the frequency of 1 rad s^−1^ was conducted finally.

### Preparation of TEM samples

HEPES solutions of Nap-GFFYGRGD and Nap-GFFYGRGD with different equiv. of Gd(III) were prepared as described above. Next, 10 μL of each sample placed on a carbon-coated copper grid and incubated for 30 seconds to allow the peptide nanostructures to adhere to the substrate, then rinsed twice with ultrapure water. The samples were then stained with a saturated uranyl acetate solution and placed in a desiccator overnight prior to analysis. Similar process was used to prepare samples of HEPES solution containing 125 μM of Gd(III) and 6 equiv. of Nap-GFFpYGRGD (750 μM) with or without phosphatase.

### Preparation of cell lysates for MRI measurements

The 3T3, HepG2, HeLa were maintained in our lab. Cells were cultured in DMEM supplemented with 10% vFBS and 100 U/mL penicillin/streptomycin at 37 °C in a humidified atmosphere of 5% CO_2_. Different cell lines were counted and then broken by a sonic oscillator. Next, 100 μL of lysates (from 2 * 10^4^ cells) of different cell lines were added into the HEPES solution containing Gd(III) and Nap-GFFpYGRGD.

### *In Vitro* MRI

*In vitro* T_1_-weighted MR images were obtained on a 1.2 T MRI system (Huantong Corporation, Shanghai, China). The parameters adopted were as follows: TR/TE = 100.0/8.8 ms, slice thickness = 1mm, 30.0 °C. Gd(III)-peptide samples were dispersed in HEPES buffer at various Gd(III) concentrations. The relaxation time values (T_1_) were also measured on the same MRI system (1.2 T) by the inversion recovery sequence. The r_1_ and r_2_ relaxivity values were determined through the curve fitting of 1/T_1_ and 1/T_2_ relaxation time (s^−1^) versus the Gd (III) concentration (mM).

### Removal of Gd(III) from water

To investigate the removal of Gd (III) from water, 10 mL of the solution containing 10 ppm of Gd(III) was prepared. Hydrogel was prepared by 125 μM of Gd(III) mixed with 6 equiv. of Nap-GFFYGRGD (Gd(III): peptide = 1:6) overnight. Then resulting hydrogel was filtered (0.45 μm) to remove the nanofibers. After filtered, the amount of Gd(III) in the filtrate was determined by Inductively Coupled Plasma-Atomic Emission Spectrometry (ICP-AES).

## Additional Information

**How to cite this article**: Hua, Y. *et al*. Gd(III)-induced Supramolecular Hydrogelation with Enhanced Magnetic Resonance Performance for Enzyme Detection. *Sci. Rep.* **7**, 40172; doi: 10.1038/srep40172 (2017).

**Publisher's note:** Springer Nature remains neutral with regard to jurisdictional claims in published maps and institutional affiliations.

## Supplementary Material

Supplementary Information
