# Supplementary material for: Gd(III)-induced Supramolecular Hydrogelation with Enhanced Magnetic Resonance Performance for Enzyme Detection
Source: Sci Rep. 2017 Jan 11;7:40172. doi: 10.1038/srep40172 (PMC5225466; doi:10.1038/srep40172)
Supplement: Supplementary Information [file srep40172-s1.pdf]

Supplementary Information

Gd(III)-induced Supramolecular Hydrogelation with Enhanced Magnetic Resonance Performance for Enzyme Detection

Yongquan Hua,[#, †] Guojuan Pu, [#,‡,§] Caiwen Ou, [†] Xiaoli Zhang, [§] Ling Wang, [§] Jiangtao Sun,[*, ‡] Zhimou Yang,[*, §] Minsheng Chen[*,†]

† Department of Cardiology, Zhujiang Hospital of Southern Medical University, Guangzhou 510280, P. R. China, [‡]School of Pharmaceutical Engineering & Life Science, Changzhou University, Changzhou 213164, China, P. R. China, [§]State Key Laboratory of Medicinal Chemical Biology, Key Laboratory of Bioactive Materials, Ministry of Education, College of Life Sciences, Nankai University, and Collaborative Innovation Center of Chemical Science and Engineering, Tianjin 300071, P. R. China

Figures.

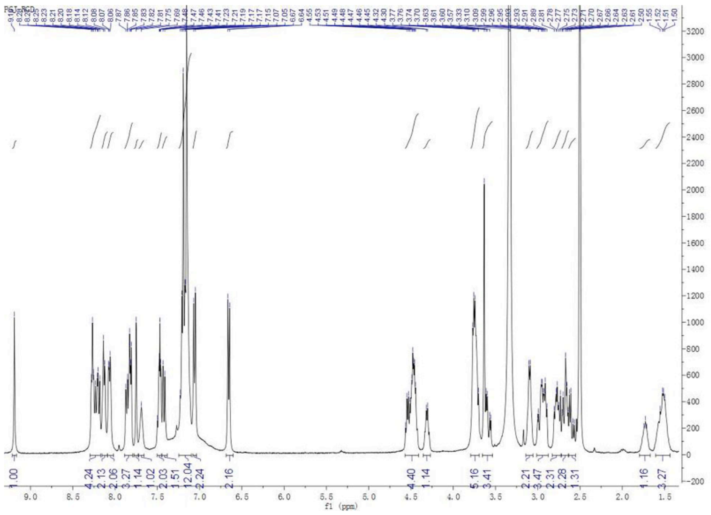

Supplementary Fig. S1 ^{1}H NMR of compound Nap-GFFYGRGD.

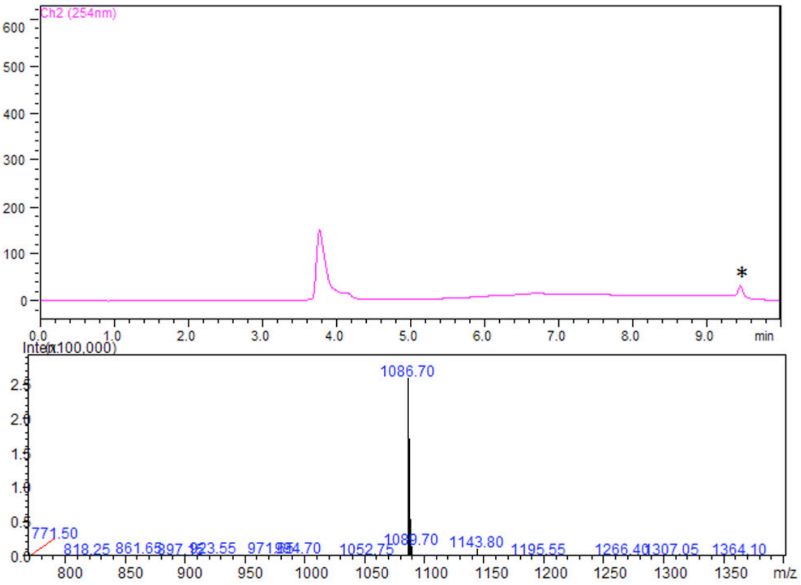

Supplementary Fig. S2 LC-MS spectra of compound Nap-GFFYGRGD (star indicates a system peak).

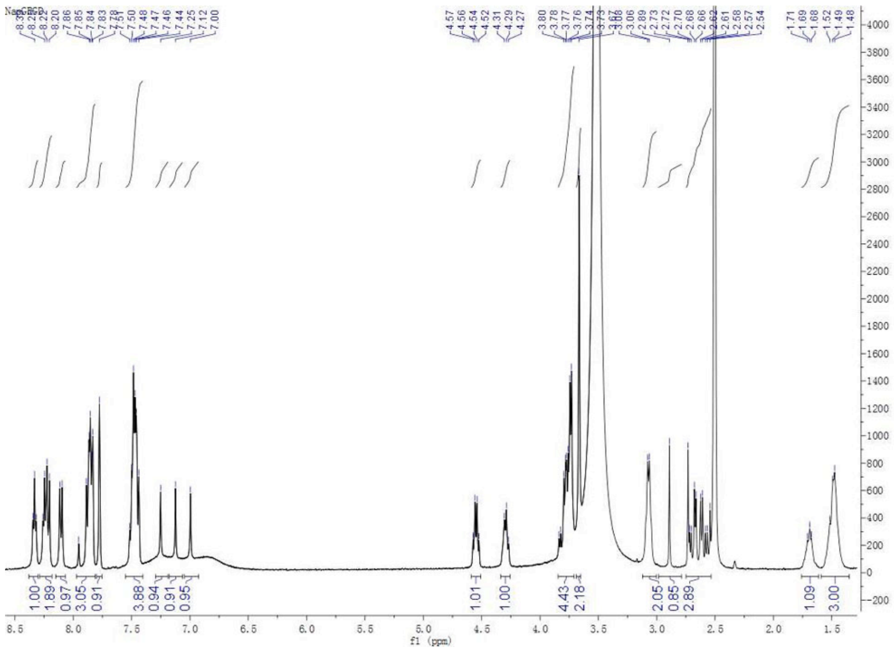

Supplementary Fig. S3 ^{1}H NMR of compound Nap-GRGD.

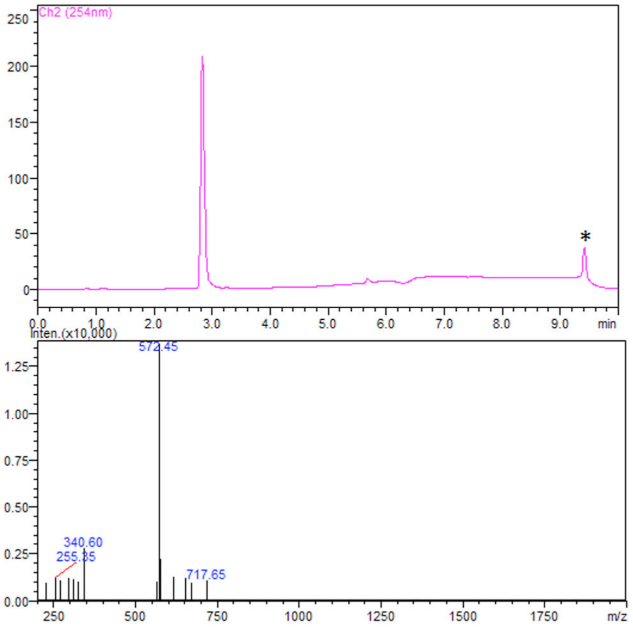

Supplementary Fig. S4 LC-MS spectra of compound Nap-GRGD (star indicates a system peak).

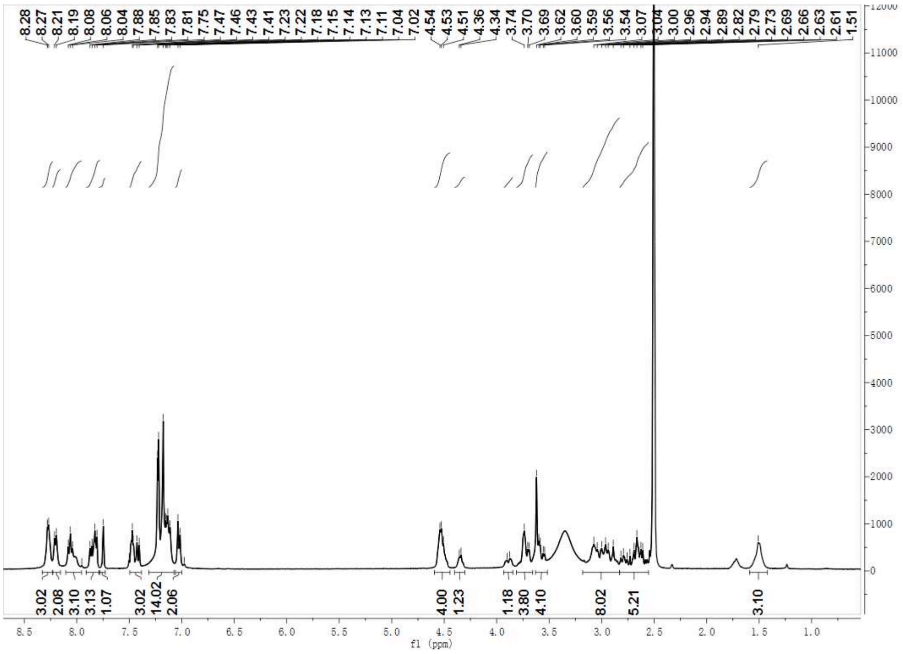

Supplementary Fig. S5 ^{1}H NMR of compound Nap-GFFpYGRGD

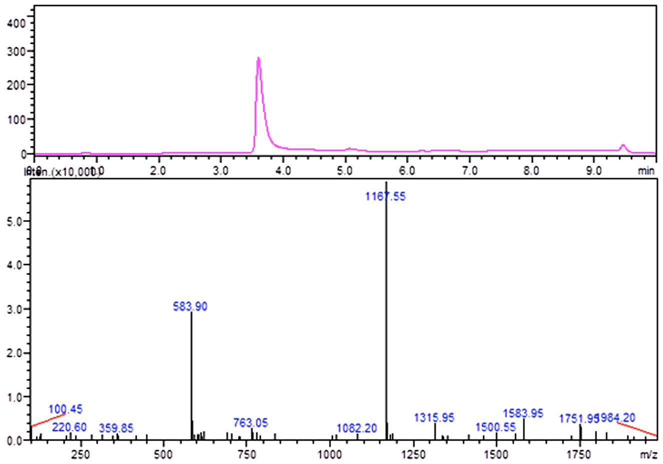

Supplementary Fig. S6 LC-MS spectra of compound Nap-GFFpYGRGD.

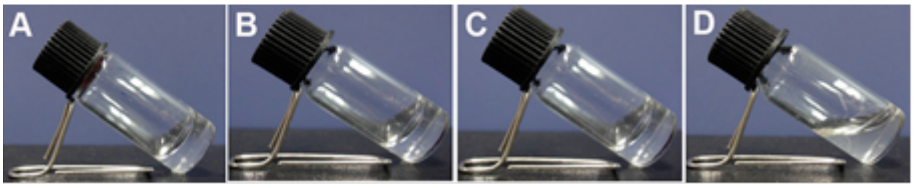

Supplementary Fig. S7 Optical images of samples containing 0.25 wt% of Nap-GFFYGRGD with A) 0.2, B) 0.1, C) 0.05 and D) 0.02 equiv. of Gd(III) in HEPES buffer solution

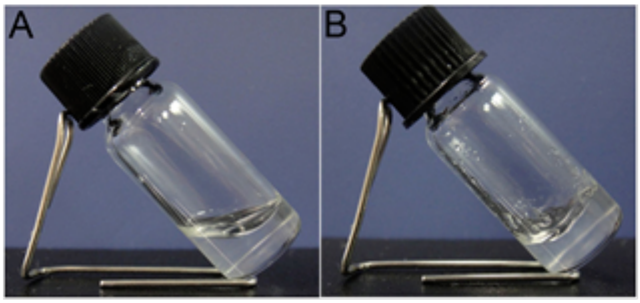

Supplementary Fig. S8 Optical images of A) a HEPES solution of Nap-GFFYGRGE (0.25 wt%, pH = 7.4), B) the gel formed by adding 0.33 equiv. of Gd(III) to the solution in A).

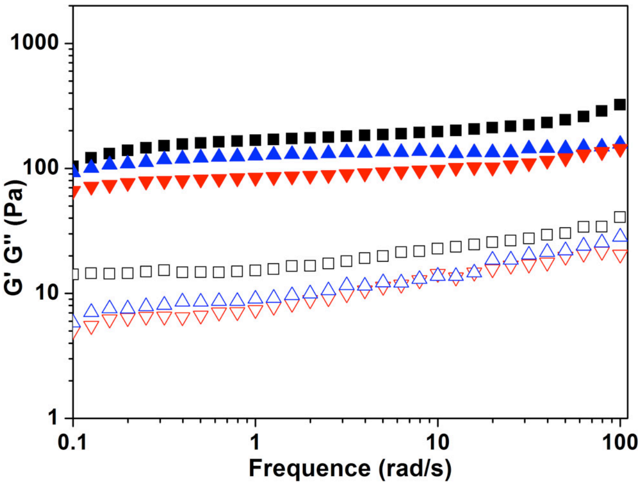

Supplementary Fig. S9 Dynamic frequency sweep of the gel formed by HEPES solution of Nap-GFFYGRGD (0.25 wt %) with 0.2 equiv. of Gd(III) (■), 0.1 equiv. of Gd(III) (▲) and 0.05 equiv. of Gd(III) (▼), (solid symbols represent elasticity(G')and the hollow ones represent viscosity (G'')).

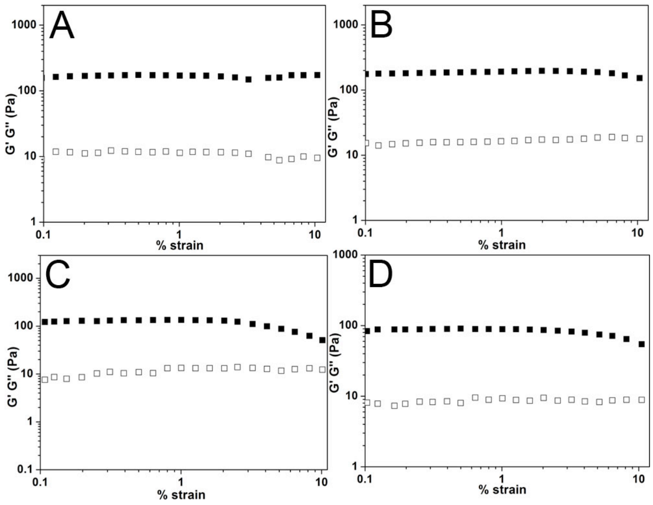

Supplementary Fig. S10 Dynamic strain sweep of the gels formed by HEPES solution of Nap-GFFYGRGD (0.25 wt %) with A) 0.33 equiv. of Gd(III), B) 0.2 equiv. of Gd(III), C) 0.1 equiv. of Gd(III), and D) 0.05 equiv. of Gd(III) at the frequency of 1 rad/s (the solid symbols represent elasticity (G') and the hollow ones represent viscosity (G'')).

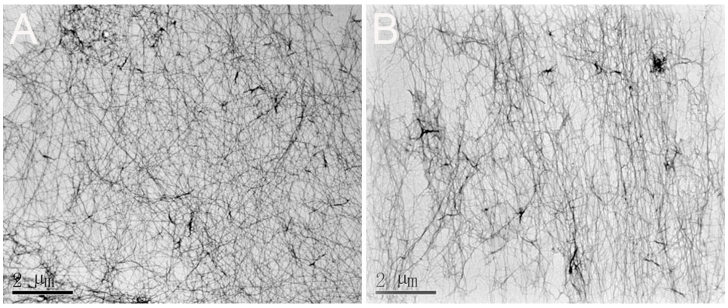

Supplementary Fig. S11 The TEM images of gels formed by A) Nap-GFFYGRGE (0.25 wt %) or B) Nap-GFFYGRGD (0.25 wt %) with 0.33 equiv. of Gd(III).

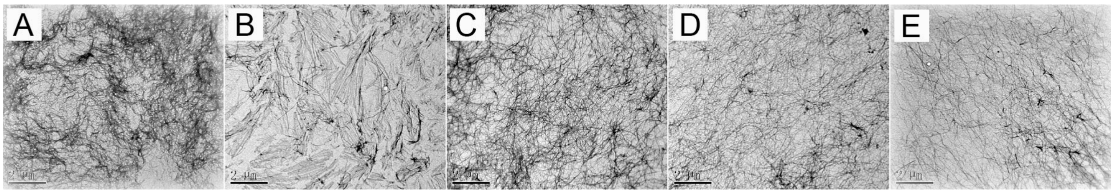

Supplementary Fig. S12 The TEM images of the solution containing 125 μM of Nap-GFFYGRGD with A) 1, B) 2, C) 3, D) 4 and E) 5 equiv. of Gd(III) in HEPES buffer solution.

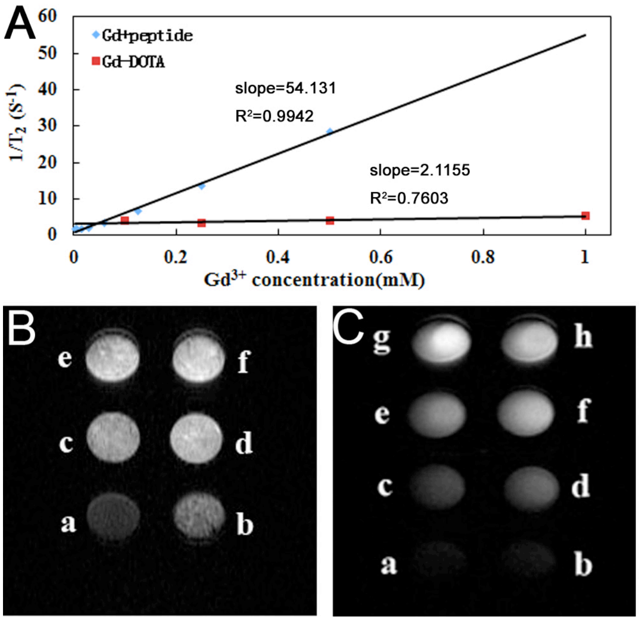

Supplementary Fig. S13 A) Plot of relaxation rate r_2 versus Gd^{3+} concentration for peptide-Gd(III) complex (Nap-GFFYGRGD : Gd(III) = 6 : 1, HEPES solution, pH = 7.4) and the Magnevist. The relaxivity value r_2 was obtained from the slope of linear fitting of the experimental data. B) 500 μM of Gd(III) in 20 mmol of HEPES buffer

(pH = 7.4), samples a-f contain increasing concentration of peptide: (a) 0, (b) 0.2, (c) 0.4 ,(d) 0.8, (e) 1, and (f) 4/3 equivalence of peptide trimer；C) samples a-h contain concentration 0.003828125、0.00765625、0.0153125、0.030625、0.06125、0.125、0.25、0.5 M of Gd(III) and contain excess peptide (6 equivalents peptide per Gd(III)), so as to ensure that >99% of Gd(III) is present as Gd (peptide)$_3$.

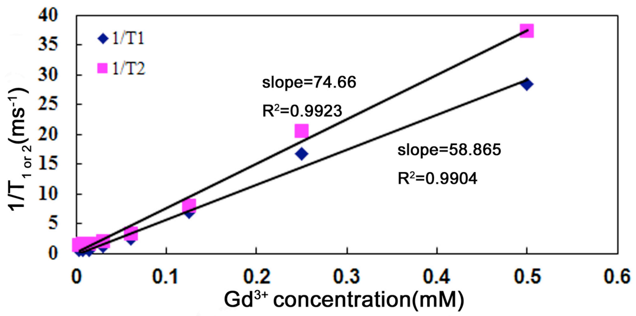

Supplementary Fig. S14 Plot of relaxation rate r_1/r_2 versus Gd^{3+} concentration for peptide-Gd(III) complex (Nap-GFFYGRGE : Gd(III) = 6 : 1, HEPES solution, pH = 7.4). The relaxivity value r_1/r_2 was obtained from the slope of linear fitting of the experimental data.

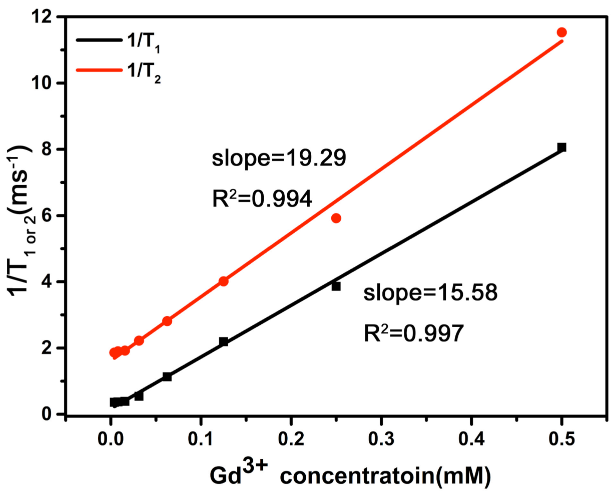

Supplementary Fig. S15 Plot of relaxation rate r_1/r_2 versus Gd^{3+} concentration for peptide-Gd(III) complex (Nap-GFFpYGRGD : Gd(III) = 6 : 1, HEPES solution, pH = 7.4) containing 1 U/mL of ALP. The relaxivity value r_1/r_2 was obtained from the slope of linear fitting of the experimental data.

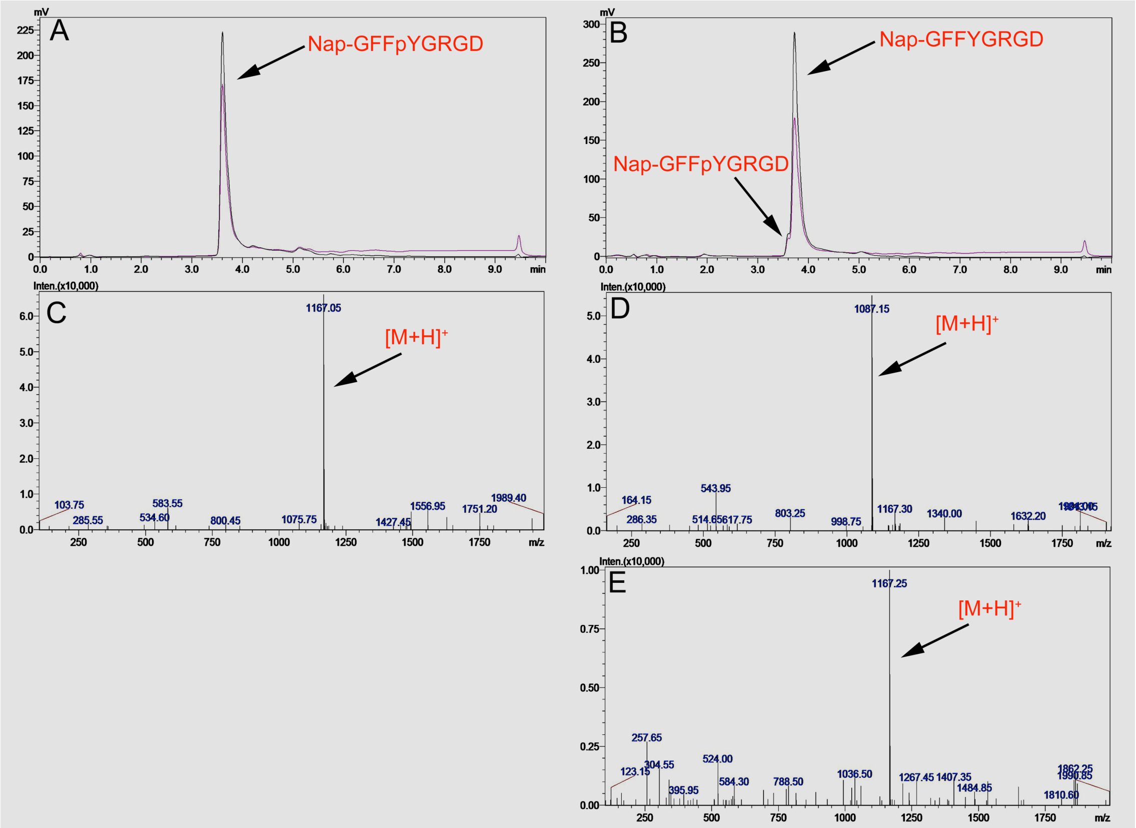

Supplementary Fig. S16 A) HPLC of Nap-GFFpYGRGD; B) HPLC of Nap-GFFpYGRGD with 1 U/mL ALP for 1h; C) MS of Nap-GFFpYGRGD in A); D) MS of Nap-GFFYGRGD cleaved from Nap-GFFpYGRGD by ALP in B), and E) MS of Nap-GFFpYGRGD un-cleaved by ALP in B).

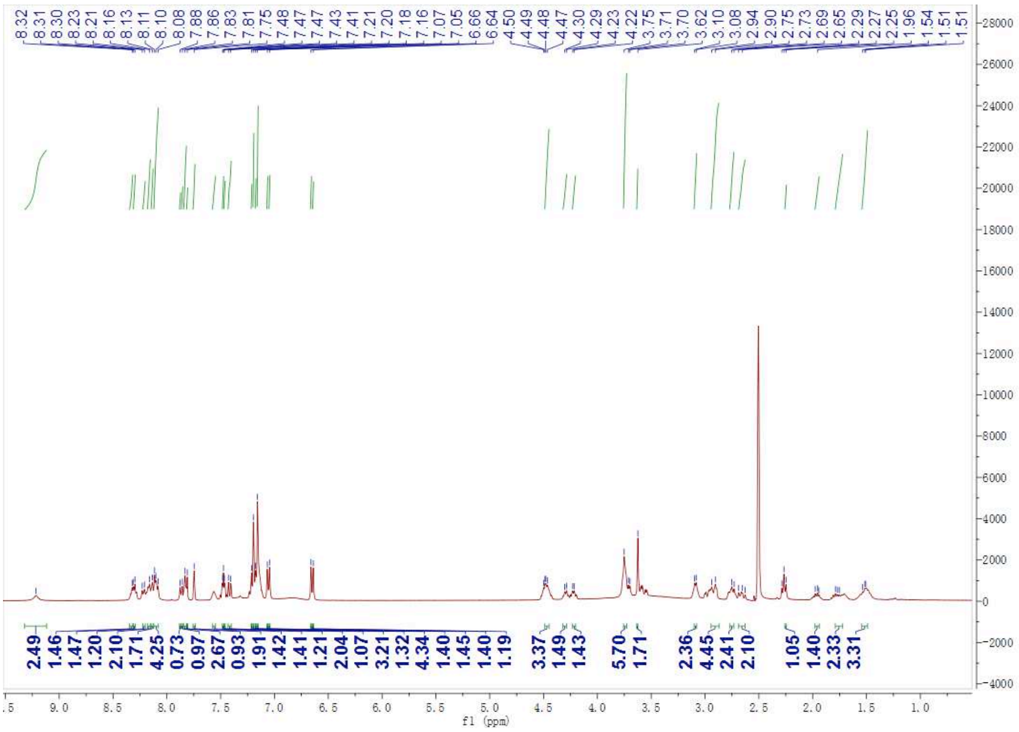

Supplementary Fig. S17 ^{1}H NMR of compound Nap-GFFYGRGE.

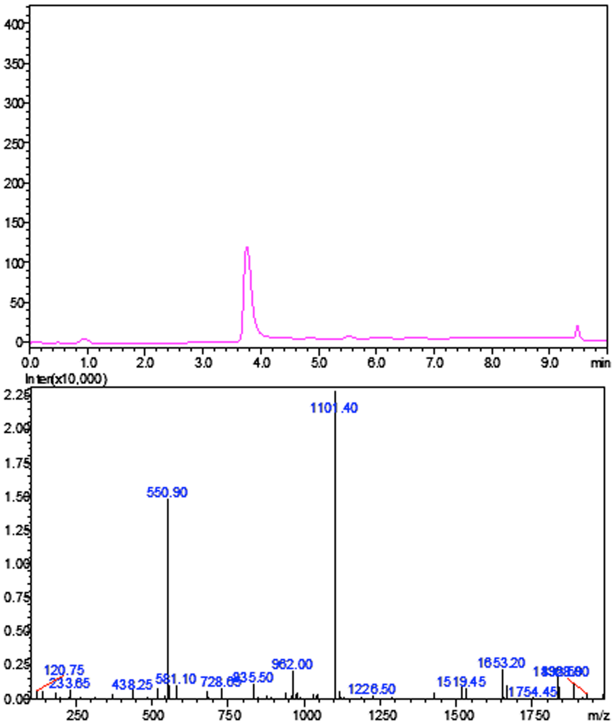

Supplementary Fig. S18 LC-MS spectra of compound Nap-GFFYGRGE.
